# Periodic health visits by primary care practice model, a population-based study using health administrative data

**DOI:** 10.1186/s12875-019-0927-6

**Published:** 2019-03-05

**Authors:** Natasha Ruth Saunders, Jun Guan, Longdi Fu, Helen Guo, Xuesong Wang, Astrid Guttmann

**Affiliations:** 1 The Hospital for Sick Children, 555 University Avenue, Toronto, Ontario M5G 1X8 Canada; 2 Department of Pediatrics, University of Toronto, Toronto, Canada; 3 ICES, Toronto, Canada; 4 Child Health Evaluative Sciences, SickKids Research Institute, Toronto, Canada; 5 Institute of Health Policy, Management and Evaluation, The University of Toronto, Toronto, Canada; 6 Dalla Lana School of Public Health, University of Toronto, Toronto, Canada

**Keywords:** Fee-for-service, Periodic health visit, Physician enrollment

## Abstract

**Background:**

The general health check, which includes the periodic health visit and annual physical exam, is not recommended to maintain the health of asymptomatic adults with no risk factors. Different funding mechanisms for primary care may be associated with the provision of service delivery according to recommended guidelines. We sought to determine how use of the periodic health visit for healthy individuals without comorbidities, despite evidence against its use, differed by primary care model.

**Methods:**

Population-based cross-sectional study using linked health and administrative datasets in Ontario, Canada, where most residents are insured for physician services through Ontario’s single payer, provincially funded Ontario Health Insurance Plan. Participants included all living adults (> 19 years) in Ontario on January 1st, 2014, eligible for the Ontario Health Insurance Plan. Primary care enrollment model was the main exposure and included traditional fee-for-service, enhanced fee-for-service, capitation, team-based care, other (including salaried), and unenrolled. The main outcome measure was receipt of a periodic health visit during 2014. Age-sex standardized rates of periodic health visits performed during the one-year study period were analyzed by number of comorbid conditions.

**Results:**

Of 10,712,804 adults in Ontario, 2,350,386 (21.9%) had a periodic health visit in 2014. The age-sex standardized rate was 6.1% (95% confidence interval [CI] 6.0, 6.1%) for healthy individuals. In the traditional fee-for-service model, the periodic health visit was performed for 55.3% (95% CI 54.4, 56.3%) of healthy individuals versus 10.2% (95% CI 10.0, 10.3%) in team-based care. Periodic health visit rates varied by primary care provider models. Traditional and enhanced fee-for-service models had higher rates across all comorbidity groups.

**Conclusions:**

Patients whose primary care physicians are funded exclusively through fee-for-service had the highest rates of periodic health visits in healthy individuals. Primary care reform initiatives must consider the influence of remuneration on providing evidence-based primary care.

## Introduction

Increasing evidence suggests general health checks, which include both the traditional annual physical exam and the periodic health visit, do not reduce morbidity and mortality for patients and is a costly service [1–3]. Insurance and care providers have reconsidered its benefit and have moved from providing less effective annual physical exams for healthy individuals towards a periodic health visit [4]. The periodic health visit focuses on preventative care, and does not necessarily include a physical examination. Physician discretion determines if a periodic health visit is warranted and it is tailored to the specific needs of each patient. The Canadian Task Force for Preventive Health Care recently commented that the traditional annual physical examination of asymptomatic adults is not supported by evidence and may result in harm. They explain there may be better value in periodic preventive visits according to age, risk and specific test intervals [5]. A recent Cochrane review [1] suggests that general health checks, including periodic health visits, are unlikely to be beneficial and are not supported based on the best available evidence. This is particularly important for healthy, low risk individuals in the `Choosing Wisely` [6] climate, where there is a focus to encourage clinicians and patients to consider reducing unnecessary tests and treatments and to make effective choices for high-quality care.

Currently, the Ontario Health Insurance Plan (OHIP), the universal government funding program for physician and hospital services in the province, funds one annual periodic health visit per patient, regardless of comorbidities. This fee code was established in November 2012, despite available evidence challenging the utility of periodic health visits. The new fee code for periodic health visits was introduced to replace the annual physical exam and was remunerated at a lower ‘price-point’ (30% less for those 18 to 65 years). Decisions around performing the periodic health visit, including for those without comorbidities, are made by the physician. Evaluation of how or why decisions for this funded but clinically unsupported service are performed has not been evaluated.

Across Ontario, a number of funding and governance models for the provision of primary care exist [7]. Details of these models are shown in Table 1. These include a traditional fee-for-service model, an enhanced fee-for-service model (Family Health Groups and Comprehensive Care Model) whereby the majority of income is through fee-for-service but incentives, premiums and bonuses are paid for eligible services to enrolled patients, capitation models where physicians receive fixed annual sums for each registered patient but are eligible for bonuses (Family Health Organizations and Family Health Networks) and a small proportion of fee-for-service payments, a team-based-care model where remuneration is a combination of capitation, bonuses, and fee-for-service (Family Health Teams), and salaried models that function independent of the number of services provided or patients served (Community Health Centres). While each model has its merits, the majority of patients are enrolled (“rostered”) in capitation or team-based care. Only a small proportion of the adult population (10%) continue to be served by practitioners who bill exclusively fee-for-service.

**Table 1:** Key features of compensation models for primary care in Ontario

	Overview	Examples of specific bonuses (not exhaustive)	% office visits PHV (2014)
Family Health Team	• Inter-professional team model • Regular and extended hours • Blended capitation model with complement-based remuneration and bonuses/incentives, or blended salary model • Ministry of health funded inter-professional teams providing primary health care (e.g. nurse practitioners, dieticians, pharmacists, social workers, psychologist, and occupational therapists).	• Similar to capitation models but the size of the capitated basket is smaller.	5.3%
Enhanced Fee-for-Service	• Comprehensive Care Management (solo practitioners) and Family Health Group Models (3+ physicians) • Primarily through fee-for-service but also eligible for specific bonuses and premiums based on patient enrolment. • Provide care to rostered patients with some after-hours care. • Limited to no professional team.	1. Patient Registration Incentive 2. Comprehensive Care Management Fee 3. After Hours Care 4. Diabetes and Heart Failure Management Incentives 5. Cumulative Preventative Care Management Payment (Bonus): Pap smears, mammograms, childhood immunizations, flu shots, colorectal screening. 6. Smoking Cessation Counselling Fee 7. Unattached Patient Fee 8. Primary Health Care of Patients with Serious Mental Illness.	6.7%
Primarily Capitation	• Family health networks and Family Health Organizations • 3+ physicians compensated mostly through capitation payments + some fee-for-service payments. • Specific bonuses & premiums based on patient enrolment, after hours premiums.	1. Patient Registration Incentive. 2. Comprehensive Care Management Fee. 3. Diabetes and Heart Failure Management Incentives. 4. Cumulative Preventative Care Management Payment (Bonus): for Pap smears, mammograms, immunizations, flu shots, colorectal screening. 5. Preventative Care Management Service Enhancement Fee (Reminder Fee): to contact patient to obtain preventative services 6. Special Payments (Premiums): eligible for all premiums in any fiscal year for: Obstetrical Deliveries, Hospital Services, Palliative Care, Office Procedures, Prenatal Care, Home Visits 7. Newborn Care Episodic Fee. 8. Smoking Cessation Counselling Fee 9. Primary Health Care of Patients with Serious Mental Illness Unattached Patient Fee	6.0%
Other (Salaried)	• Community health centres, salaried physicians, rural-northern physician group agreement, Group health Centre, Community Sponsored Agreements. • Primary care services alongside health promotion and community development programs for communities and individuals with complex needs.		4.3%
Fee-for-Service	• Traditional model. Solo. “Full-service” primary care billing. • Episodic care for patients. Some walk-in-clinics. • No professional team.		7.9%

*PHV* Periodic Health Visits

Given competing priorities of providing quality care and adequate compensation for physicians’ services, evidenced-based care delivery may differ by primary care model. Traditional fee-for-service models may lead to the over-provision of services, including periodic health visits [8]. Quality of care may suffer in such models where volume is remunerated and where interprofessional team-based care is discouraged because in order to bill, physicians must provide the service directly. Conversely, capitation models may contribute to the under-provision of services, especially for patients with multi-morbidity [8]. In the absence of additional value-based components to capitation models, this model of care has little incentive for quality based care. Bundled care models can encourage team-based approaches but may increase the quantity and intensity of physician workload as a result of increased patient volume expectations [9] Evidence for the success of pay-for-performance models in primary care has been mixed and currently in Ontario, there is no direct pay-for-performance incentive for providing the periodic health visit [8, 10–13]. Understanding physician and health system drivers of periodic health visit billing practices are important and have not been studied. The objectives of this study were to determine how use of the periodic health visit for apparently healthy individuals, despite evidence against its use, differed by primary care remuneration models. We hypothesized that models of care that had largely fee-for-service remuneration models would have the highest rates of performance of the periodic health visit in both healthy individuals and in those with comorbidities.

## Methods

This was a population-based cross-sectional study of 10 million Ontario adults through linkage of multiple population-based health databases available at ICES. Databases were linked based on each patient’s unique, encoded OHIP number and the cohort was extracted by a trained analyst. This study was approved by the Research Ethics Board at Sunnybrook Health Sciences Centre in Toronto, Canada.

### Databases

The Registered Persons Database contains demographic information for all Ontario residents who have ever received an Ontario health card number. It is updated monthly and loss of eligibility following death is accurate in 98.6% of individuals [14]. The Corporate Provider Database includes demographic, specialty, and practice location for physicians in Ontario and has 100% linkage to unique physician billing numbers and specialty codes, and the Client Agency Program Enrollment identifies the enrollment of Ontarians in a program with a specific practitioner and primary care enrollment group. 99.5% of primary care physicians who roster patients are assigned to a valid primary care model in this dataset [14]. The OHIP Claims Database identifies claims for physician services provided by physicians in Ontario. The Canadian Institute for Health Information Discharge Abstract Database and Same Day Surgery Database includes records of patients admitted to Ontario hospitals and records all same-day surgeries in Ontario hospitals, respectively.

### Study population

The study included all living adult (> 19 years) Ontario residents (39% of adult Canadians) on January 1st, 2014. Periodic health visits performed in Ontario on OHIP-eligible residents from January 1st to December 31st, 2014 were included. A two-year look-back at hospital and OHIP records was carried out to identify the number of comorbid conditions. These were assigned using the Johns Hopkins Adjusted Clinical Groups Case Mix System [15], a validated tool [15], which is based on patients’ age, gender and the diagnosis codes on patient medical records in both ambulatory (OHIP and Same Day Surgery) and inpatient care settings Discharge Abstract Database from January 2012 to December 2013. There are 32 Adjusted Diagnostic Groups. The sum of Adjusted Diagnostic Groups was used to categorize population into different numbers of comorbid conditions.

### Assignment of care provider

Eligible, rostered (enrolled) OHIP residents were identified through the Client Agency Program Enrollment database and assigned their pre-existing practitioner and primary care enrollment group (model). The models included Family Health Teams, enhanced fee-for-service, primarily capitation-based, and “other” models (e.g. salary) (Table 1). For the remaining unenrolled patients, primary care providers (PCP’s) were assigned based on the physician who provided the highest adjusted cost (adjusted for shadow billings) of the core primary care billings in OHIP from January 2012 to December 2013. These non-rostered patients were assigned to the traditional fee-for-service model as they were not formally enrolled with a particular service provider. Individuals with no primary care billings and not in the Client Agency Program Enrollment database but eligible for OHIP and living in Ontario were grouped into a “no primary care provider” group. In Ontario, PCP’s may only belong to one primary care enrolment model.

### Annual physical exam

Physician fee codes (A003, General Assessment with a diagnostic code 917 [annual health examination adolescent/adult] or K131 and K132 [periodic health visits]) billed using OHIP were used to ascertain periodic health visits and the corresponding PCP who provided the service from January to December 2014. If a patient changed his/her PCP, the PCP who performed the periodic health visit was assigned as the PCP.

### Statistical analysis

Age-sex standardization technique was used to adjust the confounding effects of differences in age-sex structure among the sub-populations being compared. The overall eligible Ontarians were adopted as the standard population, and direct age-sex standardized periodic health visit rates by sectors were calculated. A multiple variable Poisson regression model was built to test the association of primary care model and periodic health visit, adjusting for age, sex, neighbourhood income quintile, and number of comorbidities. Initial models showed there was an interaction between primary care model and comorbidity group and therefore models were stratified by the number of comorbidities. Statistical testing was carried out using SAS Enterprise Guide, version 6.1 (SAS Institute Inc., Cary, NC).

## Results

Of the 10,712,804 eligible Ontarians, 2,350,386 (21.9%) had a periodic health visit in 2014. Periodic health visit rates increased by number of comorbid conditions. Age-sex standardized rate was 6.1% for the healthy group and 25.5% for patients with > 1 comorbidity (Table 2). Periodic health visit rates varied by PCP models. Traditional and enhanced fee-for-service models had higher rates across all comorbid condition groups. There was a 3-fold standardized rate difference observed within primary care models (35.4% for traditional fee-for-service, 12.2% for other enrollment model group). Traditional fee-for-service PCPs performed the highest number of periodic health visits for the healthy adults (55.3%) (Table 2).

**Table 2:** Number and age-sex standardized rate per 100 persons who received a periodic health visit by number of comorbid conditions in Ontario in 2014

	Total				Number of comorbid conditions											
					0				1				2+			
Primary care model	# of people who received PHV	Pop^n^	Rate	95% CI	# of people who received PHV	Pop^n^	Rate	95% CI	# of people who received PHV	Pop^n^	Rate	95% CI	# of people who received PHV	Pop^n^	Rate	95% CI
Overall	2,350,386	10,712,804	21.9	(21.9, 22.0)	86,442	1,421,597	6.1	(6.0, 6.1)	149,846	993,880	15.1	(15.0, 15.2)	2,114,098	8,297,327	25.5	(25.4, 25.5)
Family Health Team	386,237	2,467,715	15.2	(15.2, 15.3)	18,332	180,653	10.2	(10.0, 10.3)	32,278	258,101	12.1	(11.9, 12.2)	335,627	2,028,961	16.2	(16.1, 16.3)
Enhanced Fee For Service	1,054,382	3,481,168	30.4	(30.3, 30.5)	31,325	189,780	16.6	(16.4, 16.7)	54,577	274,472	20.2	(20.0, 20.4)	968,480	3,016,916	32.5	(32.5, 32.6)
Primarily Capitation	567,294	2,815,694	19.7	(19.7, 19.8)	21,392	187,749	11.4	(11.3, 11.6)	40,203	263,547	15.0	(14.9, 15.2)	505,699	2,364,398	21.1	(21.1, 21.2)
Other (Salaried) Enrollment Model	11,007	85,724	12.2	(11.9, 12.4)	581	7167	7.7	(7.1, 8.4)	970	9651	9.4	(8.8, 10.0)	9456	68,906	13.1	(12.8, 13.4)
Fee For Service	331,466	962,425	35.4	(35.3, 35.5)	14,812	26,683	55.3	(54.4, 56.3)	21,818	149,784	15.3	(15.1, 15.5)	294,836	785,958	38.6	(38.4, 38.7)
No primary care provider	0	900,078	0.0	–	0	829,565	0.0	–	0	38,325	0.0	–	0	32,188	0.0	–

*PHV* Periodic health visit, *Pop*^*n*^ Population

In the multiple variable models (Table 3), patients without comorbidities in traditional fee-for-service model were 5.52 times more likely to receive periodic health visits compared with those in family health teams. Healthy individuals in an enhanced fee-for-service model also had considerably higher rates of periodic health visit compared with those in family health teams (adjusted rate ratio 1.66, 95% CI 1.63, 1.69). In patients with multiple comorbidities, those in fee-for-service and enhanced fee-for-service models had the highest rates of periodic health visits (fee-for-service: adjusted rate ratio 2.41, 95% CI 2.40, 2.42; enhanced fee-for-service: adjusted rate ratio 2.01, 95% CI 2.00, 2.02). Results were similar when stratified by those 65 years and older and those less than 65 years (Table 4). High income, female sex, and middle and older age was associated with an increased likelihood of receiving a periodic health visit. Patients in salaried models and in family health teams had the lowest likelihood of receipt of a periodic health visit across all comorbidity groups (Table 3).

**Table 3:** Rate ratios for receiving a periodic health visit by primary care model, comorbidities, and socio-demographics

*N* = 10,712,804	Number of Comorbidities		
	0	1	2+
	Adjusted* rate ratios (95% Confidence Intervals)		
Primary Care Model			
Enhanced Fee For Service	1.66 (1.63, 1.69)	1.68 (1.66, 1.70)	2.01 (2.00, 2.02)
Primarily Capitation	1.13 (1.11, 1.15)	1.23 (1.21, 1.25)	1.29 (1.29, 1.30)
Salaried Enrolment Model	0.79 (0.73, 0.86)	0.80 (0.75, 0.85)	0.84 (0.83, 0.86)
Fee For Service	5.52 (5.40, 5.64)	1.28 (1.26, 1.30)	2.41 (2.40, 2.42)
No Primary Care Physician	0.00 (0.00, 0.00)	0.00 (0.00, 0.00)	0.00 (0.00, 0.00)
Family Health Team	(reference)	(reference)	(reference)
Age (years)			
20–44	(reference)	(reference)	(reference)
45–64	1.45 (1.43, 1.47)	1.54 (1.52, 1.55)	1.46 (1.45, 1.46)
65–74	1.30 (1.26, 1.34)	1.67 (1.64, 1.70)	1.67 (1.66, 1.68)
75+	0.67 (0.64, 0.71)	1.09 (1.06, 1.12)	1.32 (1.31, 1.32)
Sex			
Female	1.11 (1.10, 1.13)	1.39 (1.38, 1.41)	1.26 (1.25, 1.26)
Male	(reference)	(reference)	(reference)
Neighbourhood Income Quintile			
1 (lowest)	(reference)	(reference)	(reference)
2	1.08 (1.06, 1.11)	1.10 (1.08, 1.12)	1.09 (1.08, 1.09)
3	1.11 (1.09, 1.14)	1.14 (1.12, 1.16)	1.13 (1.12, 1.14)
4	1.16 (1.14, 1.19)	1.22 (1.20, 1.24)	1.20 (1.19, 1.20)
5 (highest)	1.20 (1.18, 1.23)	1.30 (1.28, 1.32)	1.25 (1.25, 1.26)
Missing	0.92 (0.83, 1.02)	0.92 (0.84, 1.00)	0.93 (0.91, 0.95)

*Adjusted for age, sex, and neighbourhood income quintile

**Table 4:** Rate ratios for receiving a periodic health visit by primary care model stratified by age and number of comorbidities

N = 10,712,804	Number of Comorbidities		
	0	1	2+
	Adjusted* rate ratios (95% Confidence Intervals)		
Adults 65 years and older			
Primary Care Model			
Enhanced Fee For Service	1.09 (1.02, 1.16)	1.52 (1.46, 1.58)	1.95 (1.93, 1.96)
Primarily Capitation	0.92 (0.86, 0.99)	1.08 (1.04, 1.13)	1.24 (1.23, 1.25)
Salaried Enrolment Model	0.92 (0.73, 1.16)	0.91 (0.78, 1.06)	0.90 (0.87, 0.93)
Fee For Service	4.20 (3.88, 4.55)	1.28 (1.21, 1.35)	2.25 (2.22, 2.27)
No Primary Care Physician	0.00 (0.00, 0.00)	0.00 (0.00, 0.00)	0.00 (0.00, 0.00)
Family Health Team	(reference)	(reference)	(reference)
Adults under 65 years			
Primary Care Model			
Enhanced Fee For Service	1.72 (1.69, 1.75)	1.70 (1.68, 1.73)	2.04 (2.03, 2.05)
Primarily Capitation	1.15 (1.12, 1.17)	1.25 (1.23, 1.27)	1.32 (1.31, 1.32)
Salaried Enrolment Model	0.77 (0.70, 0.84)	0.78 (0.73, 0.84)	0.82 (0.80, 0.84)
Fee For Service	5.64 (5.52, 5.77)	1.29 (1.27, 1.31)	2.48 (2.46, 2.49)
No Primary Care Physician	0.00 (0.00, 0.00)	0.00 (0.00, 0.00)	0.00 (0.00, 0.00)
Family Health Team	(reference)	(reference)	(reference)

*Adjusted for age, sex, and neighbourhood income quintile

## Discussion

Using health and administrative data in a universal single-payer setting, this analysis demonstrates 21.9% of eligible Ontario adults and 6.1% of healthy adults received a periodic health visit in 2014. A higher rate of periodic health visits was observed with increasing number of comorbid conditions. While some variation by socioeconomic status, age, and sex was observed, there was a striking difference in the portion of adults receiving a periodic health visit by PCP model not explained by comorbidities. More than half of healthy individuals received periodic health visits in the traditional fee-for-service model, a proportion more than nine times that of the rest of the healthy population.

Evidence supports the decision for a physician to perform a physical exam should be based on medical necessity [1]. Similarly, preventative counselling and screening should be based on individual risk rather than a blanket approach to primary care delivery. Since the introduction of primary care reform, some have evaluated the impact of these reforms on patient care access [16, 17], quality [13, 18], and on drivers of evidence-based care [19, 20]. Yet, despite substantial discourse on primary care models, few studies have compared physician behaviour by payment model. One Ontario study reported differences in prevention activities between practices in the funding models. The authors report practice structure, rather than funding arrangement, was a more important factor in the delivery of evidence-based preventative care [20].

Proponents of continued use of the periodic health visit, despite the lack of evidence for its benefit in healthy individuals, assert the periodic health visit satisfies the patient’s desire to create and maintain a supportive and trusting relationship with their PCP [21]. Healthcare providers may also justify the periodic health visit using a similar rationale. However, our findings support the notion that provision of the periodic health visit to healthy patients and of evidenced-based care may be related, in part, to financial incentive and not only to a desire to provide quality care and build relationships. Disincentivizing primary care models that encourage volume-based fee-for-service payment that engender superfluous services should be considered in reforms to primary care. Not only may such volume-based fee-for-service models encourage the inappropriate provision of the periodic health visit in healthy individuals, they may hinder receipt of such visits by the elderly or those with comorbid conditions whose visits may take more time or effort by the physician due to complexity.

The relatively low rates of periodic health visits across the non-fee-for-service models in individuals with comorbidities may reflect primary preventative care that occurs at ‘sick visits’ or at visits for specific comorbidities where incentives for comprehensive care already exist. For example, some blended models in Ontario offer financial incentives for comprehensive diabetes or heart failure management. At visits for these conditions, physicians may also provide screening or counselling that might otherwise be provided at a periodic health visit in individuals without diabetes or heart failure. Thus, the need for a periodic health visit in individuals with comorbid conditions may be substituted by visits where other financial incentives are in place. Alternatively, given the more frequent health care contacts in individuals with comorbid conditions, the need for a periodic health visit to create and maintain a supportive relationship with a primary care physician may no longer be needed [21]. The low rates of periodic health visits in these non-fee-for-service models may also reflect under service in these populations as a result of a lack of financial incentive for periodic health visits.

Strengths of this study include its large sample size with inclusion of virtually all adult Ontarians eligible for a periodic health visit. This is the first province-wide evaluation of provision of periodic health visits in healthy individuals and in is important in light of the Choosing Wisely [6] campaign, and Cochrane review [1] on evidence against general health checks.

There are some limitations to this study. While a several-fold higher rate of periodic health visit in the traditional fee-for-service model was identified, causation was not established, nor was determination of whether findings are related to other factors of this care model including patient characteristics. Physician characteristics of those providing traditional fee-for-service were not studied and those providing care in this model may differ in their focus on preventative medicine, and motivation for periodic health visits may not only be financial benefit.

## Conclusions

Funding through traditional fee-for-service had the highest rates of administration of periodic health visits in otherwise healthy individuals, a practice for which there is little evidence of benefit. While individual patient and provider characteristics may be important, primary care reform initiatives must also bear in mind the influence of remuneration on delivery of evidence-based care. These results can be used to inform policy-makers about features to consider in the development and implementation of care models that support cost-effective and evidence-based primary care delivery.

## Abbreviations

OHIP: Ontario Health Insurance Plan
PCP: Primary Care Provider
